# Rare non-traumatic periprosthetic femoral fracture with features of an atypical femoral fracture: a case report

**DOI:** 10.1186/s13256-015-0590-z

**Published:** 2015-05-06

**Authors:** Takahiro Niikura, Sang Yang Lee, Yoshitada Sakai, Ryosuke Kuroda, Masahiro Kurosaka

**Affiliations:** 1 Department of Orthopaedic Surgery, Kobe University Graduate School of Medicine, 7-5-1 Kusunoki-cho, Chuo-ku Kobe, 650-0017 Japan; 2 Division of Rehabilitation Medicine, Kobe University Graduate School of Medicine, 7-5-1 Kusunoki-cho, Chuo-ku Kobe, 650-0017 Japan

**Keywords:** Atypical femoral fracture, Bisphosphonate, Dermatomyositis, Glucocorticoid, Periprosthetic fracture

## Abstract

**Introduction:**

Atypical femoral fractures have emerged as one of the potential complications of bisphosphonates during the past decade. The American Society for Bone and Mineral Research published a Task Force report on atypical femoral fractures in 2010 and a second report in 2014. Although the current definition of atypical femoral fractures in these reports excludes periprosthetic fractures, each of three published case reports describe a bisphosphonate-associated atypical femoral fracture that occurred around the stem of a total hip arthroplasty. We report a rare case of an atypical femoral fracture that occurred at the stem tip of a total hip arthroplasty that fulfills the major criteria defined by the second American Society for Bone and Mineral Research Task Force report for an atypical femoral fracture and that was associated with prolonged use of bisphosphonate.

**Case presentation:**

A 69-year-old Japanese woman with a right cementless total hip arthroplasty undertaken 44 months previously had a right femoral shaft fracture that occurred without trauma. She related that the bone fractured while she was standing, after which she fell down. Radiographs showed a noncomminuted transverse fracture located at the tip of the stem with localized periosteal thickening of the lateral cortex. The fracture was complete, extending through both cortices, and was associated with a medial spike. Her history revealed that she had been taking prednisolone to treat dermatomyositis and interstitial pneumonia for approximately 15 years. Alendronate was administered for more than 7 years. We performed open reduction and internal fixation using a locking plate with cable grip. The latest follow-up was performed 2 years after the fracture surgery. Bony union was successful. She regained the ability to walk, although her activity was limited by her comorbidities.

**Conclusions:**

Although the current definition of an atypical femoral fracture excludes periprosthetic fractures, there may be a periprosthetic fracture with the same or similar pathology as that of an atypical femoral fracture. We must be vigilant and aware of this type of fracture, especially in patients with prolonged bisphosphonate use.

## Introduction

Atypical femoral fractures (AFFs) have emerged as one of the potential complications of bisphosphonates during the past decade [1,2]. The American Society for Bone and Mineral Research (ASBMR) published a Task Force report on AFFs in 2010 [1] and a second report in 2014 [2]. Although the current definition of AFFs in these reports excludes periprosthetic fractures [1,2], each of three published case reports describe a bisphosphonate-associated AFF that occurred around the stem of a total hip arthroplasty (THA) [3-5]. We report a rare case of an AFF that occurred at the stem tip of a THA that fulfills the major criteria defined by the second ASBMR Task Force report for an AFF and that was associated with prolonged use of bisphosphonate.

## Case presentation

A 69-year-old Japanese woman with a right cementless THA undertaken 44 months previously had a right femoral shaft fracture. She recalled that she experienced acute pain in her right thigh while standing. She believed that the fracture occurred at that point in time, after which she fell down. Thus, it was not a result of trauma.

**Figure 1:**
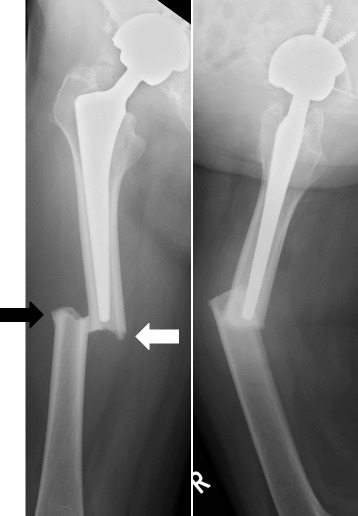
Radiographs demonstrate a periprosthetic femoral fracture with major radiographic features of an atypical femoral fracture. Note the localized periosteal thickening of the lateral cortex (black arrow) and the medial spike (white arrow).

**Figure 2:**
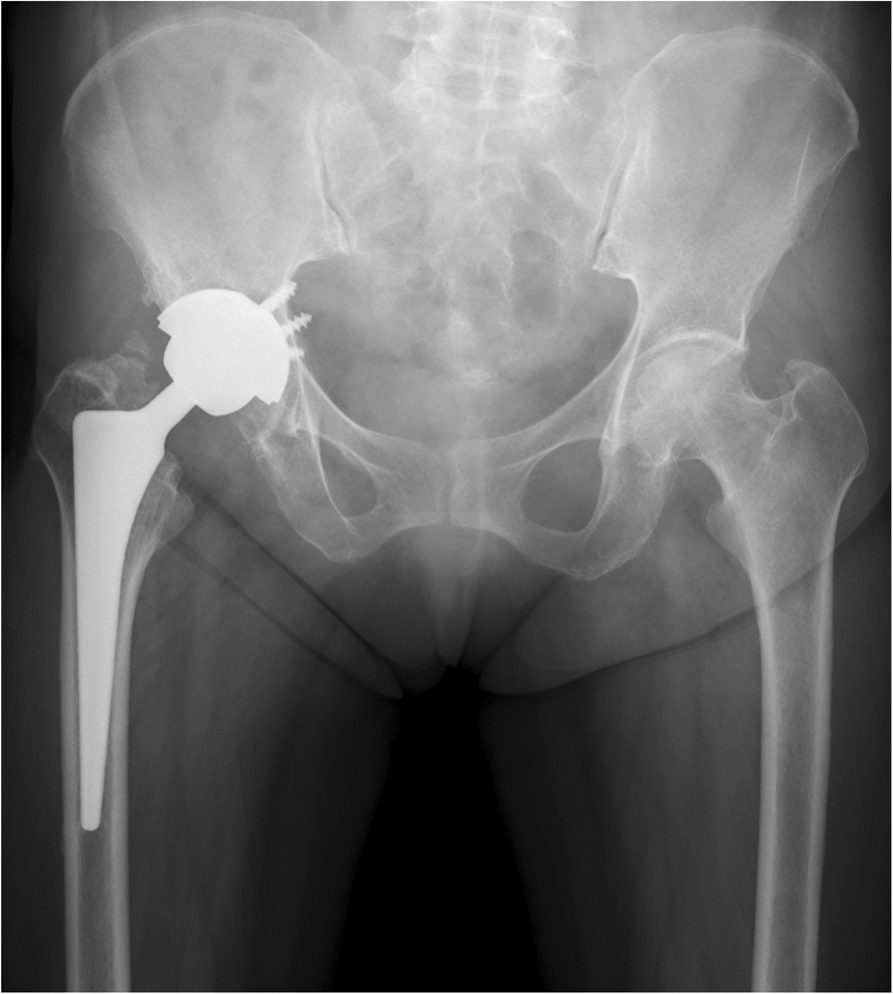
Radiograph taken at the 3-year follow-up of total hip arthroplasty (8 months before the fracture) showing no abnormal findings.

Radiographs (Figure 1) showed a noncomminuted transverse fracture located at the tip of the stem as well as localized periosteal thickening of the lateral cortex in the distal fragment. The fracture was complete, extending through both cortices, and was associated with a medial spike in the proximal fragment. There was no apparent loosening of the THA components. A radiograph taken at the 3-year follow-up of THA (8 months before the fracture) shows no abnormal findings (Figure 2).

At 15 years prior to this episode she had been diagnosed with dermatomyositis and subsequently developed interstitial pneumonia. Prednisolone and immunosuppressants (azathioprine, cyclosporine, tacrolimus) were used to treat her dermatomyositis and interstitial pneumonia. She had been on home oxygen therapy for the past 3 years. THA of her right hip had been performed to treat hip joint destruction caused by avascular necrosis of the femoral head induced by steroid use.

At admission, her height was 153.5cm, and she weighed 41kg. Her body mass index was 17.4kg/m^2^. She was a prior smoker, having stopped 21 years ago. She did not drink alcohol. She had other past medical histories. Hemi-thyroidectomy was performed for a thyroid tumor 23 years ago, and she took thyroid hormone preparation. She also had necrotizing fasciitis of the anterior cervical region that had spread from a gingival abscess 7 months ago. In addition, she underwent total hysterectomy, although she did not remember exactly when it took place. She had been taking an anti-hypertension drug and an H_2_ blocker for some time.

She had been on alendronate for at least 7 years. Her oral surgeon discontinued it 7 months prior to this fracture. It was replaced by activated vitamin D and vitamin K to treat her osteoporosis. The bone mineral density examined after the fracture of the left femoral neck was 0.644g/cm^2^, which is 82% of the young adult mean. Radiographs and magnetic resonance imaging revealed no abnormalities in her contralateral femur.

**Figure 3:**
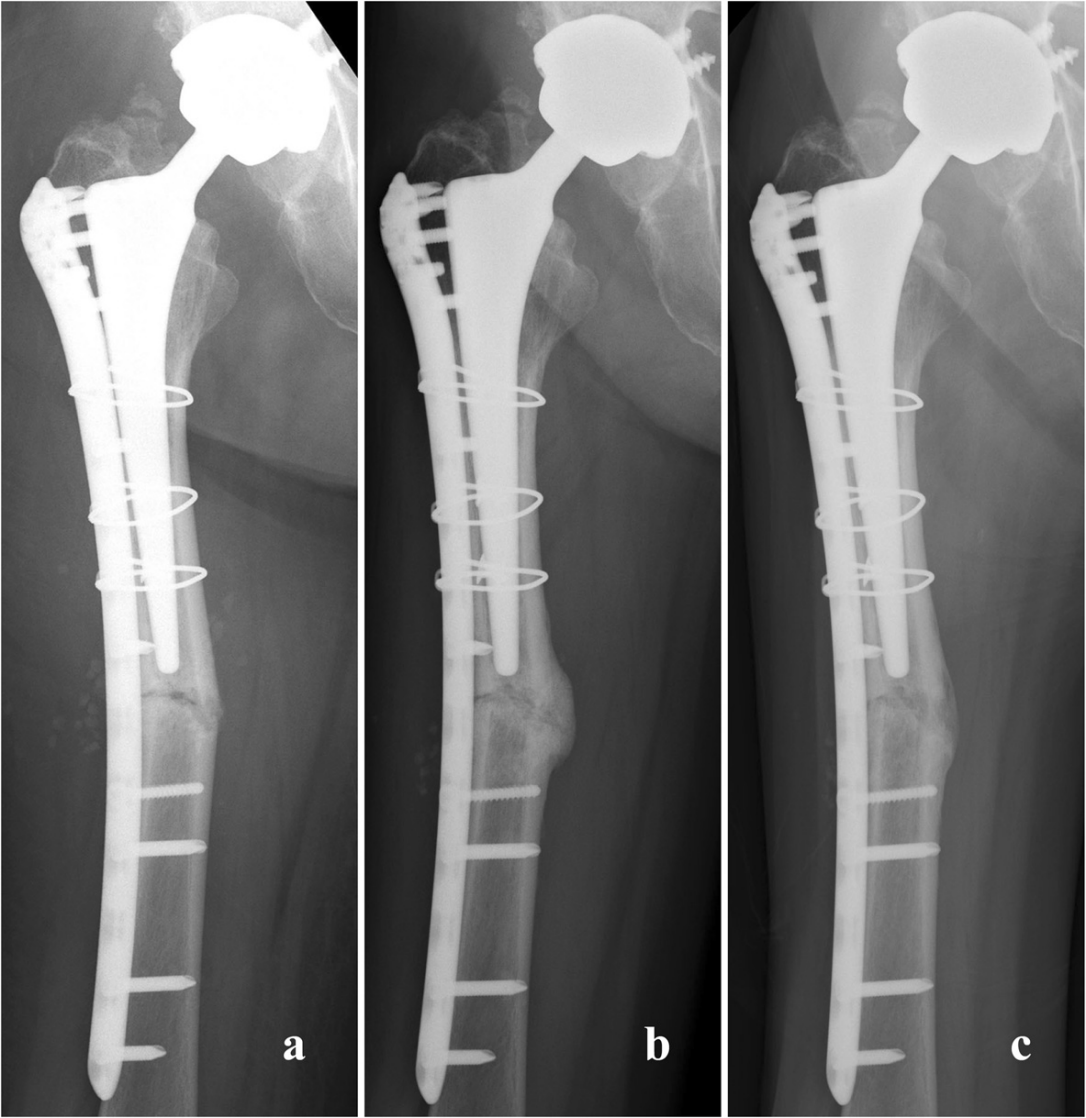
Radiographs obtained after osteosynthesis. **(a)** Just after the surgery. **(b)** At 6 months after surgery. **(c)** At 2 years after surgery.

We performed open reduction and internal fixation using a locking plate. We chose a reversed locking compression plate for the distal femur (LCP-DF; Synthes, Tokyo, Japan) for the contralateral side and an LCP cable system (Synthes). We judged that some fracture gap remained after the reduction and fixation, and so placed a small amount of β-tricalcium phosphate bone substitute to fill the gap. Autologous bone grafting was not performed. Bone healing progressed with time, and bony union was obtained 6 months after the surgery (Figure 3).

She regained the ability to walk, although her activity was limited by her comorbidities, especially interstitial pneumonia. The latest follow-up was performed 2 years after the fracture surgery. She had no pain in her right hip joint or thigh. She could walk with a cane at home and used a wheelchair outside the home. A radiographic examination revealed no displacement or loosening of the implants, and the fracture site had remodeled well (Figure 3).

## Discussion

There are three case reports describing periprosthetic femoral fractures which were associated with AFFs [3-5]. In 2009, Sayed-Noor and Sjödén [3] reported a periprosthetic femoral fracture around a cemented THA stem of a 78-year-old woman who had a 9-year history of alendronate use. The patient complained of thigh pain after a same-level fall, with localized periosteal thickening of the lateral cortex identified on a radiograph 4 months later. The fracture level was a little below the stem tip and within the cement mantle. The fracture was treated nonoperatively with no weight bearing. However, no improvement was observed, and the fracture line progressed to the medial cortex. Open reduction and internal fixation was performed with an angle-stable plating system with cable grip, similar to our operation. At the latest 5-month follow-up, radiographs showed bridging callus formation. The authors emphasized the importance of being aware of the possible correlation between long-term alendronate therapy and this insufficiency femoral fracture.

In 2011, Curtin and Fehring [4] reported three cases of periprosthetic femoral fracture around THA stems associated with prolonged bisphosphonate use. All three patients had rheumatoid arthritis and had undergone chronic medical therapy including glucocorticoids. All of the patients were women aged 52, 85, and 79 years, respectively. The bisphosphonates that had been used over time included alendronate in two patients and risedronate in one patient. A trauma history (a fall) was reported only for one patient. Cement was used for stem fixation in two patients. The fractures were incomplete, with localized periosteal thickening of the lateral cortex. The fracture levels were a little below the stem tip in one patient with a cementless stem, within the stem in a patient with a cemented stem, and a little below the stem tip and within the cement mantle in the third patient who had a cemented stem. They were treated conservatively with cessation of bisphosphonate use, subsequently being given calcium and vitamin D or teriparatide. The fractures all healed successfully. The authors noted the importance of maintaining suspicion for an AFF being responsible for a painful THA. They suggested that an AFF around a THA stem might become more common in the clinical setting, and one must be vigilant and aware of this etiology of periprosthetic hip pain.

In 2012, Cross *et al*. [5] reported a periprosthetic femoral fracture around a cemented THA stem in an 81-year-old woman who had a more than 12-year history of alendronate use. The patient presented with a 5-month history of activity-related pain in the thigh in the absence of any traumatic event. The fracture was incomplete with localized periosteal thickening of the lateral cortex. The fracture level was within the stem. The patient was treated conservatively, with cessation of alendronate use, using teriparatide and limited weight bearing. The fracture healed successfully. The authors noted that although the current definition of an AFF excludes periprosthetic fractures, this case suggested that these fractures do occur and should be considered in the differential diagnosis of patients on long-term bisphosphonates who present with thigh pain despite a well-fixed stem.

We agree with these authors and think that there may be a periprosthetic femoral fracture with similar pathology to that of an AFF. The ASBMR Task Force reports exclude periprosthetic fractures in the definition of AFF [1,2]. Hence, we cannot define this kind of fracture as an AFF. However, we believe the pathology of the current case includes the pathology of the AFF. The current case exhibited all the five major features defined by the second ASBMR Task Force report [2]. First, the fracture was not associated with trauma. Second, the fracture was a complete transverse fracture. Third, the complete fracture extended through both cortices and was associated with a medial spike. Fourth, the fracture was noncomminuted. Fifth, there was apparent localized periosteal thickening of the lateral cortex at the fracture site. In addition, the patient had a history of prolonged use of bisphosphonates and glucocorticoids, which were listed as minor features of AFFs in the prior ASBMR Task Force report [1].

Gill *et al*. [6] reported two cases of lateral insufficiency periprosthetic femoral fractures caused by osteopenia and varus angulation of the stem. There was also subsequent increased mechanical loading applied to the lateral cortex, which was seen to be extremely thin on radiographs. The current case and previously reported cases did not show malalignment of the stem accompanying the thickened lateral cortex. Therefore, we believe the pathology reported by Gill *et al*. is totally different from the current case and previously reported cases.

The second ASBMR Task Force report suggested that AFFs are stress fractures [2]. Because bisphosphonates localize in areas that are developing stress fractures, suppression of targeted intracortical remodeling by bisphosphonates at the site of an AFF is likely to impair the processes by which stress fractures normally heal [7]. The lateral cortex of the femur is known to sustain high levels of tensile stress due to bending, which may precipitate the damage in this location for AFFs [8,9]. There was a well-fixed stem in the current case, and we suspect that the tensile stress force was more concentrated on the lateral cortex at the location of the stem tip where the fracture occurred than occurs in femoral bone without a stem. We think that the location where the tensile stress force is most concentrated on the lateral cortex is variable depending on the stem design, stem alignment, or the use of cement. This may be the reason why the sites of the previously reported and current periprosthetic femoral fractures with features reflective of AFFs vary around the stem. Prolonged bisphosphonate use decreases bone remodeling [10], causes microdamage accumulation [11,12], and impairs healing of stress fractures [13,14]. We suggest that these effects of prolonged bisphosphonate use affect the incidence of periprosthetic femoral fractures with features reflective of AFFs.

The fracture healing process in the current case was uncomplicated. Adequate callus formation and remodeling were observed. We believe that cessation of alendronate intake 7 months before the fracture was instrumental in the timely remodeling, with no delay. If the contralateral side has an asymptomatic AFF, teriparatide may be used for treatment [10,15]. In our case, the contralateral side had no AFF, and we did not use teriparatide.

## Conclusions

Although periprosthetic fractures are excluded by the current definition of AFFs, there may be a periprosthetic fracture that has the same or similar pathology as an AFF. We must be vigilant and aware of this type of periprosthetic fracture, especially for patients who have a history of prolonged bisphosphonate use.

## Consent

Written informed consent was obtained from the patient for publication of this case report and accompanying images. A copy of the written consent is available for review by the Editor-in-Chief of this journal.
